# Coupling next-generation sequencing to dominant positive screens for finding antibiotic cellular targets and resistance mechanisms in *Escherichia coli*

**DOI:** 10.1099/mgen.0.000148

**Published:** 2018-01-10

**Authors:** Hélène Gingras, Bédis Dridi, Philippe Leprohon, Marc Ouellette

**Affiliations:** Centre de Recherche en Infectiologie, Université Laval, Québec, Canada

**Keywords:** antibiotic, resistance, next generation sequencing, functional cloning, drug targets, *Escherichia coli*

## Abstract

In order to expedite the discovery of genes coding for either drug targets or antibiotic resistance, we have developed a functional genomic strategy termed Plas-Seq. This technique involves coupling a multicopy suppressor library to next-generation sequencing. We generated an *Escherichia coli* plasmid genomic library that was transformed into *E. coli.* These transformants were selected step by step using 0.25× to 2× minimum inhibitory concentrations for ceftriaxone, gentamicin, levofloxacin, tetracycline or trimethoprim. Plasmids were isolated at each selection step and subjected to Illumina sequencing. By searching for genomic loci whose sequencing coverage increased with antibiotic pressure we were able to detect 48 different genomic loci that were enriched by at least one antibiotic. Fifteen of these loci were studied functionally, and we showed that 13 can decrease the susceptibility of *E. coli* to antibiotics when overexpressed. These genes coded for drug targets, transcription factors, membrane proteins and resistance factors. The technique of Plas-Seq is expediting the discovery of genes associated with the mode of action or resistance to antibiotics and led to the isolation of a novel gene influencing drug susceptibility. It has the potential for being applied to novel molecules and to other microbial species.

## Data Summary

1. The next-generation sequencing fastq files have been deposited to the sequence reads archive (SRA) under BioprojectID accession PRJNA415734. This can be accessed at www.ncbi.nlm.nih.gov/bioproject/?term=PRJNA415734 and by referring to Table S1 (available in the online version of this article) for the specific SRA accession of each sample.

**Impact Statement**

Antibiotic resistance is a major public health threat, and strategies are needed for the identification of the target(s) of novel molecules and/or the mechanisms involved in their resistance. This article describes the coupling of plasmid functional screenings and next-generation sequencing for expediting the discovery of antibiotic resistance genes and targets at the genome scale. We used *Escherichia coli* for benchmarking the approach owing to the wealth of knowledge about its metabolism and resistance determinants. Our approach allowed the identification not only of several genes reported previously to influence the susceptibility of *E. coli* to antibiotics but also of novel genes. This approach could be applied to other transformable bacteria and could refine our understanding of the mode of action of antimicrobial molecules.

## Introduction

Antimicrobial resistance is now high on the international public health agenda. In the field of antibiotic development, few novel drugs have been introduced recently in clinical settings and these new drugs are often directed at the same known drug targets [1, 2]. A number of novel approaches are being considered [3] but this will require a holistic understanding of the mode of action of drugs and microbial strategies used for resisting the action of drugs. Whole-cell phenotypic screens are currently favoured over biochemical target screens for the discovery of new compounds. The former looks for changes in cellular behaviour, morphology or survival upon screening of whole cells with large libraries of chemical compounds. It has the main advantage of revealing chemical entities that are able to penetrate cell barriers, but at the cost of knowledge about their mode of action. However, genomics can lead rapidly to the identification of drug targets of active compounds and a combination of phenotypic and target screens is now possible. Indeed, knowing the target of a lead compound can be helpful for further drug optimization.

Genomic libraries have been used to transform bacteria or parasites and to screen for genes that provide a selective advantage in the presence of drugs by acting as multicopy suppressors [4–7]. Recently, we transfected a *Leishmania* cosmid genomic library in wild-type parasites, selected them step by step with drugs, isolated the cosmids at each selection step and then characterized them by next-generation sequencing (NGS). This technique coupling library selection and NGS was termed Cos-Seq and it allowed the isolation of an unprecedented number of drug targets and resistance mechanisms in the parasite *Leishmania* [8].

We have adapted Cos-Seq to *Escherichia coli*, a bacterial species found ubiquitously in human and veterinary medicine as well as in the environment. *E. coli* can carry many antibiotic resistance genes [9], and strains carrying carbapenemases are part of the WHO pathogen priority list for antibiotic development [10]. Because of its smaller genome size, we used an *E. coli* plasmid genomic library instead of a cosmid library, which was transformed into *E. coli*. These transformants were selected independently with five different antibiotics, and plasmids isolated at each step were sequenced by NGS in a process that we named Plas-Seq. For the five drugs studied, 13 different drug targets or genes decreasing susceptibility were isolated, three that were shared between different drugs.

## Methods

### Bacterial strains, culture conditions and chemicals

Unless otherwise stated, *E. coli* strains used in this study were grown in lysogeny broth (LB; Difco) or on LB agar plates. Broth cultures were incubated at 37 °C under shaking at 250 r.p.m. for 16 h, and LB agar plates in a 37 °C incubator for 16 h. Ceftriaxone (CRO), tetracycline (TET) and trimethoprim (TMP) were purchased from Sigma Aldrich. Gentamicin (GEN) and levofloxacin (LEV) were purchased from Biobasic and Santa Cruz Biotechnology, respectively.

### Construction of Plas-Seq library and selection

*E. coli* ATCC 25922 was used as the source of genomic DNA for producing the plasmid libraries. It is a biosafety level 1, serotype O6 and biotype 1 strain that the Clinical and Laboratory Standards Institute (CLSI) recommends for antimicrobial susceptibility testing. It has a minimum inhibitory concentration (MIC) of 0.031 µg ml^−1^ to CRO and LEV and of 0.5 µg ml^−1^ to TET, TMP and GEN. Genomic DNA was extracted from *E. coli* ATCC 25922 using a Wizard genomic DNA purification kit (Promega) and nebulized to 2–5 kb fragments according to the manufacturer’s instruction (Nebulizers; ThermoFisher). DNA fragments of 2–5 kb were size-selected from agarose gel, end-repaired and purified. The Plas-Seq library was generated by cloning the purified 2–5 kb fragments into the pZErO-2 vector (ThermoFisher) before electroporation into TOP10 *E. coli*. The *E. coli* TOP10 cells transformed with the genomic library were selected step by step, starting with an antibiotic pressure corresponding to 0.25× the MIC and up to 2× MIC. No clones were obtained when Plas-Seq selection was pushed to 4× MIC. Indeed, we found that to be successful Plas-Seq requires small increments in antibiotic concentrations to avoid the loss of genes conferring more subtle phenotypes. The Plas-Seq selection began by incubating the transformants at 37 °C for a duration of 16 h in 5 ml LB medium supplemented with 0.25× MIC of the appropriate antibiotic, before the extraction of the plasmids using a GenElute plasmid miniprep kit (Sigma Aldrich). These extracted plasmids were then transformed back into *E. coli* TOP10 and plated onto five large LB agar plates supplemented with 0.25× MIC of the appropriate antibiotics. We found that this additional transformation step was useful to decrease the background of false positive clones whose resistance is due to the acquisition of spontaneous mutations in the chromosome of the host during the Plas-Seq selection rather than originating from the plasmids harboured by the clones. After overnight incubation of the plates at 37 °C, colonies were scraped off and their plasmids extracted using a GenElute plasmid miniprep kit. The bulk of plasmids extracted was used for generating the NGS libraries (see below) but a 50 ng aliquot was used for transforming *E. coli* TOP10 cells to initiate the second cycle of Plas-Seq at 0.5× MIC of the appropriate antibiotic. The cycle of plasmid extraction and retransformation was done at 0.25×, 0.5×, 1× and 2× MIC. The *E. coli* TOP10 cells transformed with the genomic library were passaged in parallel in the absence of drug to control for the enrichment of genes unrelated to antibiotic pressure. For each antibiotic, the plasmids extracted at the four antibiotic concentrations (0.25×, 0.5×, 1× and 2×), as well as the plasmids extracted at each passage for the unselected (control) cells, were sequenced by NGS. The Plas-Seq procedure was done in biological duplicates.

### Plasmid purification for Illumina sequencing

Genomic DNA was removed from plasmid extractions by digestion with Plasmid-Safe ATP-Dependent DNase (Epicentre) following the manufacturer’s instructions. Purified plasmid DNA was quantified using a QuantiFluor dsDNA System staining kit and Quantus fluorometer (Promega). Illumina Nextera XT sequencing libraries were prepared according to the manufacturer’s instructions. The size distribution of Nextera XT libraries was validated using an Agilent 2100 Bioanalyzer and High Sensitivity DNA chips (Agilent Technologies). Sequencing libraries were quantified using a QuantiFluor dsDNA System staining kit and Quantus fluorometer. These were sequenced using an Illumina HiSeq2500 system (101 nt paired-end sequencing) at a final concentration of 8 pM. The NGS data has been deposited to the sequence reads archive (SRA) under BioprojectID accession PRJNA415734; sample accessions are indicated in Table S1.

### Genome coverage and quality control

Sequencing reads from each sample were aligned independently with the genome of *E. coli* ATCC 25922 (GenBank assembly accession GCA_000743255.1) using the BWA software [11]. The maximum number of mismatches was four, the seed length was 32 and two mismatches were allowed within the seed. Files in BAM format were processed with the SAMStat (version 1.08) software (http://samstat.sourceforge.net/) to confirm sequence quality and for mapping statistics. Each sample yielded between 2 and 4 million reads. BEDTools (version 2.21.0) (http://bedtools.readthedocs.io/en/latest/) was used to convert BAM files to BED files for the visualization of read depth and genome coverage using the SignalMap software (Roche NimbleGen).

### Gene enrichment analysis

The detection of genes enriched in the Plas-Seq screens relied on the Trinity software version 2.1.1 [12], which includes all third-party tools required for the analysis. Gene abundance within samples was quantified using the kallisto software [13]. Clusters of genes significantly enriched by drug selection were retrieved with edgeR [14] using the default parameters (false discovery rate ≤0.001). Gene clusters were then plotted according to the median-centred log_2_ fragments per kilobase per million mapped reads (FPKM) values using R scripts included in the Trinity package. To confine analysis to the most likely significant hits, only genes with a log_2_-fold change ≥3 were retained. For these genes, the variation in FPKM between the selection step responsible for maximum enrichment and the baseline level was computed and converted to the BED format for genome-wide visualization using SignalMap. The plasmid fold-enrichment was computed by extracting the mean FPKM ratio from genes on enriched plasmids, and normalized to the control sample passaged in absence of antibiotic.

### Functional validation and MIC determination

Genes of interest for functional validation were chosen from the list of enriched genes based on the combination of fold enrichment and gene annotation, with genes the most enriched and those whose function suggested they could influence susceptibility to antibiotics being prioritized. Plasmids that were the most enriched and thus the most abundant could often be recovered by picking up random colonies from plates (e.g. plates from the 2× MIC CRO sample). For less enriched plasmids that could not be recovered on plates, we simply amplified the genes of interest by PCR using genomic DNA derived from *E. coli* ATCC 25922. Genes of interest were amplified using primers listed in Table S2 and cloned in the multiple cloning site of the pZErO-2 vector. The gene *marC* was also cloned in its antisense orientation as a control. The ligation products were transformed into *E. coli* TOP10 and ATCC 25922, and MICs were determined for CRO, GEN, LEV and TMP by agar dilution and for TET by macrodilution in triplicates and according to the CLSI procedure (https://clsi.org/standards/products/microbiology/documents/m07/).

### CRISPRi knock-down and quantitative RT-PCR (qRT-PCR)

Plasmids pdCas9-bacteria and pgRNA-bacteria used for the knock-down of *rob* were obtained from Addgene (no. 44249 and 44251). Constructs expressing guide RNAs for *rob* were generated as described previously [15]. To validate our CRISPRi knock-down, we performed qRT-PCR as described previously [16]. All qRT-PCR data were normalized according to the amplification signal of *tuf* mRNA. For specific primers see Table S2.

### Inactivation of *yebV*

Targeted gene deletion of *yebV* was guided by homologous recombination in *E. coli* EL250. A PCR cassette covering the *KAN* gene and including the flippase recombination site was amplified from the pKD4 plasmid (Addgene no. 45605) as described by Datsenko and Wanner [17]. The PCR primers (Table S2) included ∼50 bp of flanking sequences derived from the *yebV* locus. The PCR cassette was electroporated into *E. coli* EL250, and the transformants were spread onto plates containing 50 µg kanamycin ml^−1^. The flippase was then activated by the addition of arabinose, and gene deletion was confirmed by PCR using primers listed in Table S2.

## Results

**Fig. 1.**
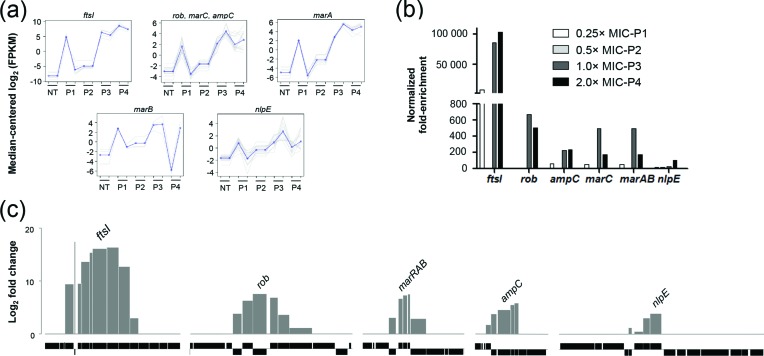
Plas-Seq identification of loci implicated in CRO resistance. (a) Plots of gene clusters sharing similar CRO Plas-Seq profiles recovered by gradual selections from which we identified a resistance gene. Pale grey lines represent individual genes, and dark grey lines denote the average profile per cluster. Gene abundance is expressed on the y-axis as log_2_-transformed FPKM values centred to the median FPKM. Samples are ordered on the abscissa according to the selection procedure [Non treated (NT); 0.25× MIC (P1), 0.5× MIC (P2), 1× MIC (P3) and 2× MIC (P4)]. Gene abundance for the two biological replicates is also shown. ‘Staircase’ patterns are due to differences in gene abundance at baseline between the replicates. (b) Fold enrichment (relative to the P0 baseline level) of the resistance gene identified at each increment in CRO concentration as normalized to the drug-free control. (c) Gene enrichment for each plasmid enriched after CRO selection and characterized functionally. Log_2_-transformed maximal variation in abundance compared with the non-treated baseline is shown. For each plasmid, the gene responsible for the resistance phenotype is indicated. Grey bars represent enriched genes. Black bars underneath represent operons (each box corresponding to a gene) surrounding the enriched loci.

The antibiotic-sensitive *E. coli* strain ATCC 25922 was used as the source of genomic DNA, which was cloned in the pZErO-2 plasmid. This library was transformed in TOP10 *E. coli* cells, and more than 320 000 clones with an average insert size of 2.1 kb were obtained, leading to a library with close to 150× genome coverage. The plasmids isolated from the library were sequenced by NGS, and the genome was well represented (Fig. S1). The *E. coli* TOP10 cells transformed with the genomic library were subjected to Plas-Seq selection using antibiotic concentrations corresponding to 0.25×, 0.5×, 1× and 2× the MIC. The Plas-Seq procedure was performed with five different classes of antibiotics including CRO, GEN, LEV, TET and TMP. As an example, the Plas-Seq output for CRO consisted of 11 different plasmids containing between one and seven genes that were enriched upon selection and revealed by NGS (Table S3). At each passage from 0.25× to 2× MIC of CRO, we observed a gradual increase of specific genomic regions as exemplified by reads abundance or fold enrichment (Fig. 1a, b). All the 48 genomic loci enriched during selection with the five antibiotics are listed in Table S3. We tested 15 out of these 48, of which 13 were shown to directly influence susceptibility to antibiotics (Table S3). These 13 loci are shown for CRO in Fig. 1(c) as well as for the four other antibiotics in Fig. S2.

### Isolation of specific genes

The next step required pinpointing the genes responsible for the phenotype among the plasmids revealed by Plas-Seq. Of the 11 plasmids that were enriched while using CRO as the selection drug (Table S3), six could be isolated and retransformed into *E. coli* and five out of six were found to increase the MIC to CRO (Tables 1 and S3). We failed to isolate the remaining plasmids or have not tested their role in resistance, but other gene products highlighted by Plas-Seq and not tested experimentally may also have a role in modulating susceptibility to CRO. Each of the five plasmids isolated and validated for increasing the MIC of CRO had between four and eight genes (Table 1 and Fig. 1c), and we tested the role of several of these genes individually. This revealed six individual genes whose overexpression increased the MIC to CRO. These include a penicillin binding protein (FtsI), the AmpC β-lactamase, a NlpE lipoprotein, two components of the *mar* operon (MarC and MarAB) and the Rob transcriptional factor (Table 1). The plasmids were enriched at each step of selection with the highest enrichment observed at either 1× or 2× MIC (Fig. 1b). The *ftsI*-containing plasmid was the one most enriched as determined by sequence reads (Fig. 1), and the *ftsI* gene was also the one displaying the strongest phenotype for CRO (Table 1). The Plas-Seq screen selected with GEN led to 12 plasmids (Table S3); we could isolate two, and both of them increased the MIC to GEN upon overexpression. The specific resistance determinants within these two plasmids were the lipoprotein NplE (which was also highlighted by the CRO screen) and a hypothetical protein (DR76_3007), YebV (Fig. S2). The product of the *yebV* gene has the Pfam motif DUF1480 of unknown function and is part of a family of enterobacterial proteins of about 80 amino acids in length. When LEV was used in the Plas-Seq screen, seven plasmids were enriched (Table S3); two were isolated, and both decreased susceptibility to LEV (Table 1). These genes coded for the transcriptional regulators *soxS* and *rob*, respectively (Fig. S2). A TET screen also led to the same two transcriptional regulators in addition to a third one encoded by *sidA* (Tables 1 and S3). For TMP, we enriched for four plasmids (Table S3), two of which were studied but only one, encoding for the FolA drug target, proved to contribute to resistance in TOP10 cells (Table 1). All MIC measures were first performed in TOP10 cells, the recipient strain of our Plas-Seq screen, but similar if not stronger phenotypes were observed when genes were tested in *E. coli* ATCC 25922 whose genome was used for generating the plasmid genomic library (Table 1). Only three of the genes whose overexpression increased survival in TOP10 cells did not lead to a phenotype in ATCC 25922 (Table 1).

**Table 1.** Genomic loci enriched in the Plas-Seq screens and genes responsible for the resistance phenotype

Drug	Plasmid*	Fold enrichment	Gene start	Gene stop	Genomic position	Resistant gene entry	Gene name	Gene product	Fold resistance	
									TOP10	ATCC 25922
CRO	1	102961	DR76_2592	DR76_2599	2764297. .2773353	DR76_2596	*ftsI*	Peptidoglycan synthase FtsI	4×	8×
	2	985	DR76_2505	DR76_2510	2666131. .2671027	DR76_2506	*rob*	Right origin-binding protein	2×	4×
	3	491	DR76_3325	DR76_3329	3540651. .3542008	DR76_3329	*marC*	MarC integral membrane protein	2×	1×
	3	491	DR76_3325	DR76_3329	3540651. .3542008	DR76_3327/DR76_3326	*marA* and *marB*	Multiple antibiotic resistance proteins	2×	1×
	4	281	DR76_2220	DR76_2223	2375197. .2378094	DR76_2221	*ampC*	Beta-lactamase	2×	4×
	5	106	DR76_2706	DR76_2709	2894751. .2896439	DR76_2709	*nlpE*	Lipoprotein NlpE	2×	1×
										
GEN	1	7277	DR76_3002	DR76_3009	3214843. .3222301	DR76_3007	*yebV*	Hypothetical protein	2×	4×
	2	6206	DR76_2705	DR76_2716	2894138. .2902771	DR76_2709	*nlpE*	Lipoprotein NlpE	2×	1×
										
LEV	1	330375	DR76_2505	DR76_2509	2666131. .2669603	DR76_2506	*rob*	Right origin-binding protein	2×	2×
	2	252538	DR76_2120	DR76_2124	2270218. .2276377	DR76_2121	soxS	Regulatory protein SoxS	2×	2×
										
TET	1	37626	DR76_2505	DR76_2509	2666131. .2669603	DR76_2506	*rob*	Right origin-binding protein	2×	4×
	2	20050	DR76_2120	DR76_2124	2270218. .2276377	DR76_2121	*soxS*	Regulatory protein SoxS	2×	2×
	3	3906	DR76_2926	DR76_2932	3143332. .3149938	DR76_2929	*sdiA*	Regulatory protein SdiA	2×	2×
										
TMP	1	406354	DR76_2556	DR76_2564	2722303. .2729487	DR76_2559	*folA*	Dihydrofolate reductase	16×†	16×†
	2	187	DR76_2507	DR76_2508	2666704. .2668901	DR76_2506	*rob*	Right origin-binding protein	1×	2×

∗The full list of enriched plasmids can be found in Table S2.

†This fold increase in MIC is meeting the EUCAST breakpoints for clinical resistance.

### Multidrug resistance

Several screens with different drugs led to the isolation of plasmids with similar gene contents. This included the genes coding for the transcription regulators Rob and SoxS and the lipoprotein NplE (Table 1). We further tested the role of these gene products and of other genes isolated by the Plas-Seq screens to assess whether they could indeed influence the susceptibility to multiple drugs. Only two of the genes tested, *fstI* and *ampC*, were specific to one of the drugs tested, CRO (Table 2), while *marC*, *marRAB*, *rob*, *nplE*, *yebV*, *soxS*, *sidA* and *folA* decreased the susceptibility against more than one antibiotics used in this investigation (Table 2). The *marC* transcriptional regulator increased the MIC to the five drugs (Table 2). Given this striking result, the *marC* gene was also cloned in its antisense orientation as a control and, as expected, this did not change the MIC to any of the five antibiotics tested (Table 2).

**Table 2.** Cross-resistance phenotype for genes enriched by Plas-Seq

Strain	MIC (µg ml^−1^)				
	CRO*,†	GEN*,†	LEV*,†	TET†,‡	TMP*,†
TOP10	0.13	0.50	0.016	1.00	0.25
pFF6§	0.13	0.50	0.016	1.00	0.25
*ftsI*||	0.50	0.50	0.016	1.00	0.25
*marC*||	0.25	1.00	0.031	2.00	0.50
*marC|| (antisense)*	0.13	0.50	0.016	1.00	0.25
*marAB||*	0.25	0.50	0.031¶	2.00¶	0.25
*ampC||*	0.25	0.50	0.016	1.00	0.25
*rob||*	0.25	0.50	0.031	2.00	0.25
*nlpE||*	0.25	1.00	0.031	2.00	0.25
*yebV||*	0.25	1.00	0.031¶	1.00	0.25
*soxS||*	0.25¶	0.50	0.031	2.00	0.50¶
*sdiA||*	0.25	0.50	0.016	2.00	0.50¶
*folA||*	0.13	0.50	0.031	1.00	4.00

*MIC measured by agar dilution.

†Genes selected with specific antibiotics are underlined.

‡MIC measured by macrodilution.

§The pFF6 vector (KAN resistance marker) was use as a control since the pZErO-2 plasmid (KAN resistance marker) is a suicidal vector.

||Gene expressed in pZErO-2 plasmid.

¶These genes were enriched <eightfold in these samples and at early selection steps.

### Further analysis of *yebV* and *rob*

**Fig. 2.**
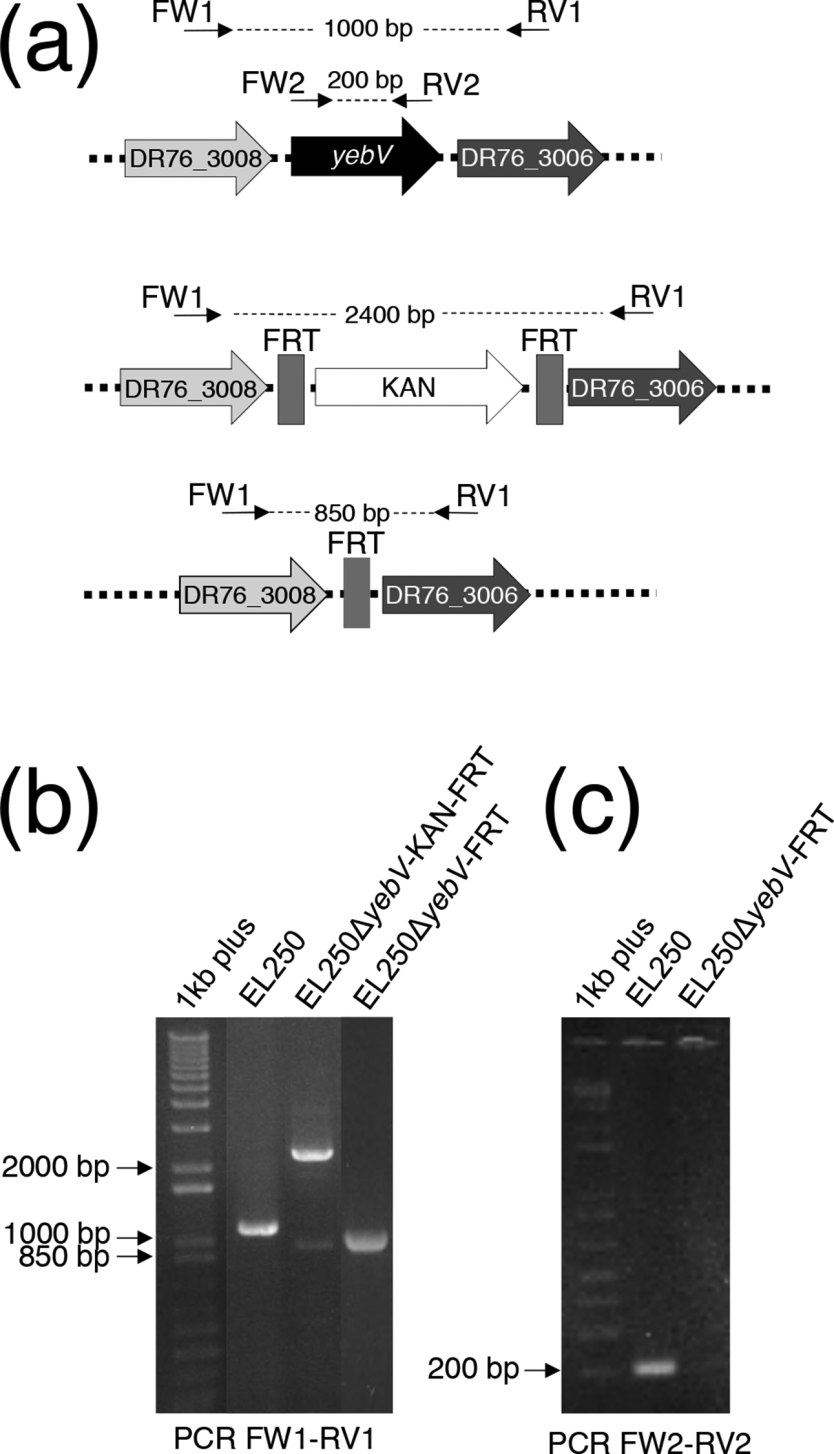
Inactivation of *yebV* in *E. coli* EL250. (a) Schematic representation of *yebV* and its manipulation in *E. coli* EL250. The location of primers FW1, RV1, FW2 and RV2 as well as the expected size of the product amplified is indicated. (b) Validation using PCR primers FW1 and RV1 of the replacement of *yebV* with KAN along with the Flippase recognition target site (EL250Δ*yebV*-KAN-FRT) and of the removal of the KAN cassette after activating the flippase using arabinose (EL250Δ*yebV*-FRT). (c) Validation of *yebV* deletion in *E. coli* EL250 using the internal PCR primers FW2 and RV2. 1 kb plus, 1 kb plus DNA ladder.

**Fig. 3.**
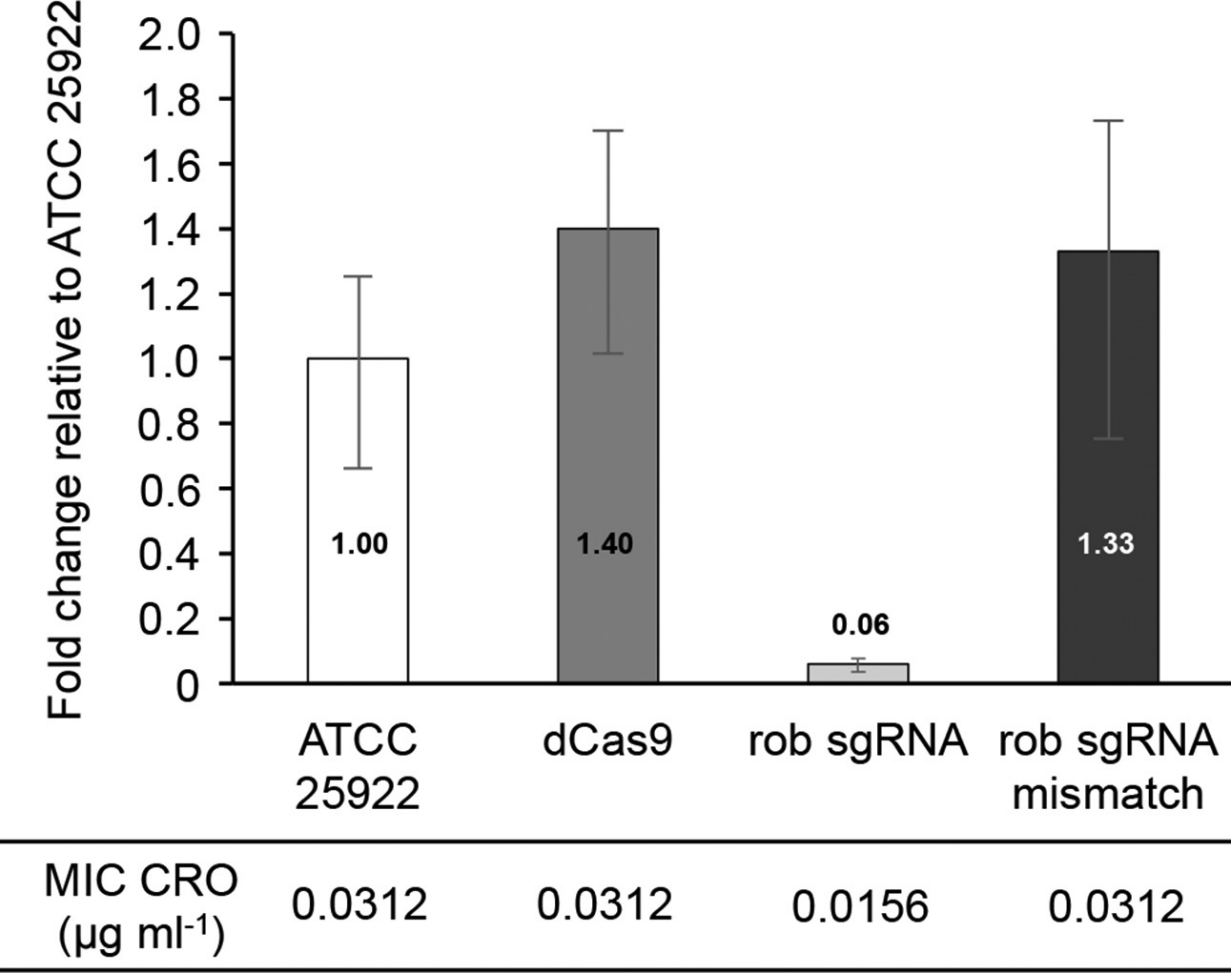
CRISPRi knock-down of the *E. coli rob* gene. Quantitative RT-PCR showing relative *rob* expression compared with that in wild-type *E. coli* ATCC 25922. dCas9, *E. coli* ATCC 25922 expressing a catalytically dead version of the Cas9 nuclease; rob sgRNA, *E. coli* ATCC 25922 expressing a catalytically dead version of the Cas9 nuclease and a single guide RNA against the *rob* gene; rob sgRNA mismatch, *E. coli* ATCC 25922 expressing a catalytically dead version of the Cas9 nuclease and an inactive version of the single guide RNA against the *rob* gene. All qRT-PCR data were normalized according to the amplification signals of the housekeeping *tuf* mRNA. MICs for CRO measured by agar dilution are shown for all strains. Error bars indicate the standard deviation for the triplicate measurements.

The phenotype conferred by the genes shown in Tables 1 and 2 resulted from their overexpression because of the multicopy nature of the plasmids. Most genes isolated were also already known to have a role in resistance to the antibiotic used for selection. Still, some of the cross-resistance such as *folA* and LEV was novel. Also, one gene, *yebV*, had never been reported to decrease susceptibility to antibiotics. It led to cross-resistance to CRO, GEN and LEV, a phenotype observed both in TOP10 and ATCC 25922 (Table 1). We generated a strain inactivated for *yebV* using the highly efficient chromosome engineering system in *E. coli* strain EL250 [17, 18]. The successful deletion of *yebV* (Fig. 2) indicated that the gene was not essential and these cells were not more susceptible to GEN, CRO or LEV. Thus, only overexpression of *yebV* and not its inactivation resulted in a noticeable effect on the MIC of GEN, CRO or LEV. To test whether this was general or specific to *yebV*, we also targeted the transcriptional factor *rob*. Instead of using a gene knockout strategy, we used the CRISPR interference (CRISPRi) technique to knock-down the expression of *rob*. CRISPRi uses a catalytically dead Cas9 along with a guide RNA which interfere with transcription in bacterial and eukaryotic cells [15, 19]. As verified by quantitative RT-PCR, we successfully knocked down the expression of *rob* when a perfect match guide RNA was used but not when the guide had two mismatches at its 3′ end just upstream of the protospacer adjacent motif (PAM) (Fig. 3). In contrast to *yebV*, cells with less *rob* mRNA had a phenotype and were more susceptible to CRO (Fig. 3).

## Discussion

We have adapted a functional gene overexpression screen coupled to NGS to *E. coli*. This allowed the rapid screening of five antibiotics and pinpointing of possible targets and genes decreasing susceptibility. Obviously, considerable work has already been done with *E. coli* and the five drugs tested but, nonetheless, we identified a role for a new gene, *yebV*. Several other enriched plasmids were not tested (Table S3), and it is possible that genes never before associated with resistance could indeed contribute to this phenotype. The overexpression of *yebV* led to reduced susceptibility not only to GEN but also to CRO and LEV (Table 2). The phenotype is only seen upon overexpression as the knock-out of *yebV* (Fig. 2) did not lead to increased drug sensitivity. Further work is warranted for understanding how YebV influences susceptibility to antibiotics.

Plas-Seq led to the isolation of drug targets including FtsI for CRO and FolA for TMP. Mutations in *ftsI* have been associated with increased resistance to β-lactam antibiotics in *Haemophilus* spp. [20], and the accumulation of mutations in *ftsI* can lead to CRO resistance [21]. While mutations have been described, overexpression of *ftsI* as a resistance mechanism to CRO seems to be novel. Overexpression of the dihydrofolate reductase gene *folA* was already known to contribute to TMP resistance [22]. It is salient to point out that *folA* overexpression led to the highest level of resistance (16×) and isolation of this gene using overexpression strategies seems to be frequent using a number of antifolates [4, 8]. The outer membrane lipoprotein NlpE was isolated with both CRO and GEN screens (Table 1). NplE is a known activator of systems involved in cell wall homeostasis, and its overexpression was shown to activate multidrug efflux pumps in *E. coli* leading to a decreased susceptibility to various antibiotics [23]. NplE, however, does not seem to confer a phenotype in all genetic backgrounds as exemplified here for ATCC 25922 (Table 1). Similarly, overexpression of *sidA* increases the MIC to several antibiotics by regulating the AcrAB effux pump [24] but this is the first time to our knowledge that it has been associated with TET and cross-resistance to CRO (Table 2), a phenotype consistent with *sidA* overexpression observed in *E. coli* cells resistant to ceftazidine [25].

The *ampC* gene was first isolated in a mutant screen in *E. coli* selecting for low penicillinase activity [26]. Promoter up-mutations leading to the overexpression of *ampC* in *E. coli* lead to β-lactam resistance [27], and transmissible plasmids having acquired *ampC* have the capacity to confer resistance to CRO and other broad-spectrum cephalosporins [27].

The related transcriptional factors MarA, SoxS and Rob play a central role in *E. coli* for surviving against antibiotic pressure and these factors have been shown to interact [28–30]. It is thus not surprising that these genes were isolated several times in our Plas-Seq screens using different classes of antibiotics targeting either the cell wall (CRO), translation (TET), DNA synthesis (LEV) or folate metabolism (TMP). The multiple antibiotic resistance (*mar*) locus [31] contributes to resistance. This locus contains two divergently expressed operons (*marC* and *marRAB*) [32]. The role of *marRAB* in resistance is well established but, in contrast to the results presented in Table 1, the *marC* gene of *E. coli* was not shown to contribute to multidrug resistance [33]. Our results have been repeated several times, and overexpression of *marC* decreased the susceptibility of *E. coli* to the five antibiotics tested (Table 2). At this point, we cannot explain this discrepancy; possibly, differences in the recipient strains could be involved. It is salient to point out that overexpression of *marC* had no detectable phenotype in ATCC 25922 (Table 1).

We have shown that Plas-Seq can lead to the identification of several genes whose overexpression decreases susceptibility to antibiotics. This could be applied to novel molecules to discover mode of action or potential resistance mechanisms. The changes in MIC were often subtle and within the experimental deviation of the techniques used but these are supported by triplicate measurements, by the fact that the phenotypes were nullified when the genes were cloned in their antisense orientation, and by the role in susceptibility to antibiotics previously reported for most of the genes identified. While not all targets can be isolated, because either their overexpression is toxic or is dominant negative, it is clear that target overexpression can often lead to increased survival under antibiotic pressure and Plas-Seq could be helpful to expedite their isolation. In our hands the overexpression of *marC* decreased the susceptibility of *E. coli* to the five antibiotics tested but it was only isolated in the CRO screen. This suggests that we could further fine tune Plas-Seq. We used biological duplicates, a strategy that proved useful in our related Cos-Seq approach [8] to decrease the number of false positive. Despite this, genes selected by one antibiotic sometimes conferred cross-resistance (Table 2) apparently without significant cross-enrichment (Tables 1 and S3) but for some genes this is explained by the ≥eightfold enrichment cut-off that we used to focus on the most highly enriched plasmids (see Methods). This Plas-Seq approach could be applied to other transformable bacteria and could expedite a better understanding of the mode of action of antimicrobial molecules.

## Data bibliography

1. Gingras H, Dridi B, Leprohon P Ouellette M. Sequence Reads Archive PRJNA415734 (2017).

## Supplementary Data

232 Supplementary File 1
