# Corrigendum: Coupling next-generation sequencing to dominant positive screens for finding antibiotic cellular targets and resistance mechanisms in *Escherichia coli*

**DOI:** 10.1099/mgen.0.000894

**Published:** 2022-10-14

**Authors:** Hélène Gingras, Bédis Dridi, Philippe Leprohon, Marc Ouellette

**Affiliations:** ^1^​ Centre de Recherche en Infectiologie, Université Laval, Québec, Canada

## Full-Text

In the published version of this article, in Table 1, it was stated that the gene responsible for the gentamycin resistance was yebV, and in Table 2 that yebV contributed to resistance to CRO, GEN, and LEV. This finding was incorrect and the article should have stated that the gene responsible for the gentamycin resistance was yebW and that it was yebW (not yebV) that contributed to resistance to CRO, GEN, and LEV.

Additionally, in the published article Fig. 2b was a composite figure and the data should have been repeated on a single gel. The correct Fig. 2 and its updated legend is found below.

**Fig. 2.**
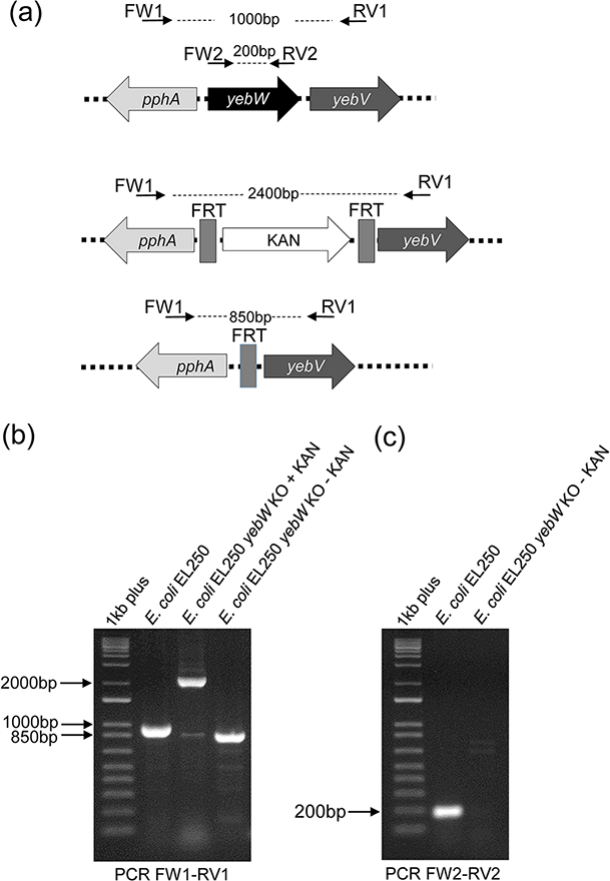
Inactivation of *yebW* in *E. coli* EL250. (**a**) Schematic representation of *yebW* and its manipulation in *E. coli* EL250. The location of primers FW1, RV1, FW2 and RV2 as well as the expected sizes of the product amplified are indicated. (**b**) Validation using PCR primers FW1 and RV1 of the replacement of *yebW* with KAN along with the Flippase recognition target site (yebW KO +KAN) and of the removal of the KAN cassette after activating the flippase using arabinose (*yebW* KO - KAN). (**c**) Validation of *yebW* deletion in *E. coli* EL250 using the internal PCR primers FW2 and RV2. 1 kb plus, 1 kb plus DNA ladder.

The authors apologize for any inconvenience caused.

