# Supplementary material for: Coupling next-generation sequencing to dominant positive screens for finding antibiotic cellular targets and resistance mechanisms in Escherichia coli
Source: Microb Genom. 2018 Jan 10;4(2):e000148. doi: 10.1099/mgen.0.000148 (PMC5857375; doi:10.1099/mgen.0.000148)
Supplement: Supplementary File 1 [file mgen-4-148-s001.pdf]

Supplementary Tables

Table S1. Sequence reads archive accession numbers for the Plas-Seq samples in this study.

SRA accession	Sample Number	Sample Name	Sample Type	Organism	Strain
SAMN07833963	sample1	CRO_025MIC_R1	Plas-Seq	Escherichia coli	ATCC 25922
SAMN07833964	sample2	CRO_025MIC_R2	Plas-Seq	Escherichia coli	ATCC 25922
SAMN07833965	sample3	CRO_05MIC_R1	Plas-Seq	Escherichia coli	ATCC 25922
SAMN07833966	sample4	CRO_05MIC_R2	Plas-Seq	Escherichia coli	ATCC 25922
SAMN07833967	sample5	CRO_MIC_R1	Plas-Seq	Escherichia coli	ATCC 25922
SAMN07833968	sample6	CRO_MIC_R2	Plas-Seq	Escherichia coli	ATCC 25922
SAMN07833969	sample7	CRO_2MIC_R1	Plas-Seq	Escherichia coli	ATCC 25922
SAMN07833970	sample8	CRO_2MIC_R2	Plas-Seq	Escherichia coli	ATCC 25922
SAMN07833971	sample9	TMP_025MIC_R1	Plas-Seq	Escherichia coli	ATCC 25922
SAMN07833972	sample10	TMP_025MIC_R2	Plas-Seq	Escherichia coli	ATCC 25922
SAMN07833973	sample11	TMP_05MIC_R1	Plas-Seq	Escherichia coli	ATCC 25922
SAMN07833974	sample12	TMP_05MIC_R2	Plas-Seq	Escherichia coli	ATCC 25922
SAMN07833975	sample13	TMP_MIC_R1	Plas-Seq	Escherichia coli	ATCC 25922
SAMN07833976	sample14	TMP_MIC_R2	Plas-Seq	Escherichia coli	ATCC 25922
SAMN07833977	sample15	TMP_2MIC_R1	Plas-Seq	Escherichia coli	ATCC 25922
SAMN07833978	sample16	TMP_2MIC_R2	Plas-Seq	Escherichia coli	ATCC 25922

SAMN07833979	sample17	GEN_025MIC_R1	Plas-Seq	Escherichia coli	ATCC 25922
SAMN07833980	sample18	GEN_025MIC_R2	Plas-Seq	Escherichia coli	ATCC 25922
SAMN07833981	sample19	GEN_05MIC_R1	Plas-Seq	Escherichia coli	ATCC 25922
SAMN07833982	sample20	GEN_05MIC_R2	Plas-Seq	Escherichia coli	ATCC 25922
SAMN07833983	sample21	GEN_MIC_R1	Plas-Seq	Escherichia coli	ATCC 25922
SAMN07833984	sample22	GEN_MIC_R2	Plas-Seq	Escherichia coli	ATCC 25922
SAMN07833985	sample23	GEN_2MIC_R1	Plas-Seq	Escherichia coli	ATCC 25922
SAMN07833986	sample24	GEN_2MIC_R2	Plas-Seq	Escherichia coli	ATCC 25922
SAMN07833987	sample25	LEV_025MIC_R1	Plas-Seq	Escherichia coli	ATCC 25922
SAMN07833988	sample26	LEV_025MIC_R2	Plas-Seq	Escherichia coli	ATCC 25922
SAMN07833989	sample27	LEV_05MIC_R1	Plas-Seq	Escherichia coli	ATCC 25922
SAMN07833990	sample28	LEV_05MIC_R2	Plas-Seq	Escherichia coli	ATCC 25922
SAMN07833991	sample29	LEV_MIC_R1	Plas-Seq	Escherichia coli	ATCC 25922
SAMN07833992	sample30	LEV_MIC_R2	Plas-Seq	Escherichia coli	ATCC 25922
SAMN07833993	sample31	LEV_2MIC_R1	Plas-Seq	Escherichia coli	ATCC 25922
SAMN07833994	sample32	LEV_2MIC_R2	Plas-Seq	Escherichia coli	ATCC 25922
SAMN07833995	sample33	TET_025MIC_R1	Plas-Seq	Escherichia coli	ATCC 25922
SAMN07833996	sample34	TET_025MIC_R2	Plas-Seq	Escherichia coli	ATCC 25922
SAMN07833997	sample35	TET_05MIC_R1	Plas-Seq	Escherichia coli	ATCC 25922

SAMN07833998	sample36	TET_05MIC_R2	Plas-Seq	Escherichia coli	ATCC 25922
SAMN07833999	sample37	TET_MIC_R1	Plas-Seq	Escherichia coli	ATCC 25922
SAMN07834000	sample38	TET_MIC_R2	Plas-Seq	Escherichia coli	ATCC 25922
SAMN07834001	sample39	TET_2MIC_R1	Plas-Seq	Escherichia coli	ATCC 25922
SAMN07834002	sample40	TET_2MIC_R2	Plas-Seq	Escherichia coli	ATCC 25922

Table S2. List of PCR primers used in this study.

Gene/Locus of interest	Primer name (restriction enzyme)	Sequence (5'-3') (restriction enzyme binding sites in bold)	Application
rob	rob_FW (KpnI)<br>rob_RV (BamHI)	GGC**GGTACC**GATCGTTACCATTTTTATCTATTACACGAGCAA<br>GGC**GGATCC**TTAACGACGGATCGGAATCAG	Subcloning in pZERO-2
ftsI	ftsI_FW<br>ftsI_RV	ATATGAAAGCAGCGGCGAAAACGCA<br>TTACGATCTGCCACCTGTCCC	Subcloning in pZERO-2
marC	marC_FW (BamHI)<br>marC_RV	CAC**GGATCC**AATTAGTTGCCCTGGCAAGTAATTAGTTGCA<br>TTAATGATACGTTTTAATGATTTCCAGGATGCCG	Subcloning in pZERO-2
marA-marB	marA_FW (BamHI)<br>marB_RV	CAC**GGATCC**AATGACGATGTCCAGACGCAATACTGA<br>CTACATAGCGTGTTGATTATAATAGGGCACG	Subcloning in pZERO-2
ampC	ampC_FW<br>ampC_RV	ATATGGGTTTTCTACGGTCTGGCTG<br>TTACTGTAGAGCGTTGAGAATCTGCCAG	Subcloning in pZERO-2
nlpE	nlpE_FW<br>nlpE_RV	CACGGATCCAATGGTGAAAAAAGCGATAGTGACAGCG<br>TTACAGCCCCAAACTACTGCAATC	Subcloning in pZERO-2
yevB	yebV_FW<br>yebV_RV	ATATGGAGAGCACGAAGATGAAAACCAGT<br>TCAGGCAAGACGCATAATCCAGG	Subcloning in pZERO-2
soxS	soxS_FW (KpnI)<br>soxS_RV (BamHI)	GGC**GGTACC**GATGTCCCATCAGAAAATTATTCAGGATC<br>GGC**GGATCC**CTACAGGCGGTGGCGATAATC	Subcloning in pZERO-2
sdiA	sdiA_FW<br>sdiA_RV	ATATGCAGGATACGGATTTTTTCAGCTGG<br>TCAAATTAAGCCAGTAGCGGCCG	Subcloning in pZERO-2
folA	folA_FW<br>folA_RV	ATATGATCAGTCTGATTGCGGCGTTA<br>TTACCGCCGCTCCAGAATCTCAA	Subcloning in pZERO-2
rob	rob-sgRNA_FW	TGACCTTCCAGCCAGATTAAGTTTTAGAGCTAGAAATAGCAAGTTAAAATAAGGC	CRISPRi
rob	rob_sgRNA_missmatch_FW	TGACCTTCCAGCCAGATTCCGTTTTAGAGCTAGAAATAGCAAGTTAAAATAAGGC	CRISPRi
Universal RV primer CRISPRi	Universal_RV	ACTAGTATTATACCTAGGACTGAGCTAGC	CRISPRi
rob	rob_qRT-PCR_FW<br>rob_qRT-PCR_RV	CCCAGAGCTACTCCTGTTCG<br>TCGTGCCAGAACTGATAACG	qRT-PCR
tuf	tuf_FW<br>tuf_RV	GTGGGAAGCGAAAATCCTG<br>CCAGTACAGGTAGACTTCTG	qRT-PCR
yebV	pKD4_yebV_FW<br>pKD4_yebV_RV	TGCGATCGGACTGGTCGTACCACAACCGGCAGCTAATGTGTAGGCTGGAGCTGCTTC<br>ACAAATAACCCGCCATGCCGACGGGTTCTTTTTGGACATATGAATATCCTCCTTA	Knock-out
yebV	Outside_yebV_FW<br>Outside_yebV_RV	CAGTGGCGGATGATGTATTG<br>CCGGCAGATGTCTTTATGGT	Knock-out
yebV	Inside_yebV_FW<br>Inside_yebV_RV	TGGAGAGCACGAAGATGAAA<br>CGGGTACGGTAAAGGACAGA	Knock-out

Table S3. Genomic loci enriched in the Plas-Seq screens.

Drug	Plasmid	Fold enrichment	Gene start	Gene stop	Genomic position	Resistant gene entry[#]	Gene name	Gene product	Fold resistance
CRO	1	102961	DR76_2592	DR76_2599	2764297..2773353	DR76_2596	*ftsI*	peptidoglycan synthase ftsI	4x
	2	985	DR76_2505	DR76_2510	2666131..2671027	DR76_2506	*rob*	right origin-binding protein	2x
	3	491	DR76_3325	DR76_3329	3540651..3542008	DR76_3329	*marC* and	marC integral membrane protein	2x
	3	491	DR76_3325	DR76_3329	3540651..3542008	DR76_3327/ DR76_3326	*marRAB*	multiple antibiotic resistance	2x
	4	281	DR76_2220	DR76_2223	2375197..2378094	DR76_2221	*ampC*	beta-lactamase	2x
	5	106	DR76_2706	DR76_2709	2894751..2896439	DR76_2709	*nlpE*	lipoprotein NlpE	2x
	6	1813	DR76_4206	DR76_4211	4429071..4432380	Not found			
	7	1461	DR76_1266	DR76_1270	1375105..1377056	Not found			
	8	1089	DR76_3811	DR76_3813	4013932..4016103	Not found			
	9	603	DR76_2022	DR76_2022	2155561..2159589	Not tested			
	10	587	DR76_362	DR76_364	395592..402754	Not found			
	11	522	DR76_3446	DR76_3446	3680350..3683874	DR76_3446	*nifJ*	pyruvate:ferredoxin oxidoreductase	1x
GEN	1	7277	DR76_3002	DR76_3009	3214843..3222301	DR76_3007	*yebV*	hypothetical protein	2x
	2	6206	DR76_2705	DR76_2716	2894138..2902771	DR76_2709	*nlpE*	lipoprotein NlpE	2x
	3	173	DR76_84	DR76_87	98222..100813	Not tested			
	4	620	DR76_465	DR76_468	523193..529716	Not found			
	5	615	DR76_851	DR76_853	941341..942650	Not found			
	6	385	DR76_2048	DR76_2048	2186608..2186991	Not tested			
	7	2124	DR76_1729	DR76_1731	1848740..1853189	Not found			
	8	320	DR76_1953	DR76_1953	2093599..2094783	Not tested			
	9	335	DR76_1226	DR76_1226	1..115	Not tested			
	10	1268	DR76_3560	DR76_3562	3803043..3805411	Not tested			
	11	1142	DR76_3503	DR76_3506	3742500..3747606	Not tested			
	12	1268	DR76_1612	DR76_1616	1738833..1741001	Not tested			
LEV	1	330375	DR76_2505	DR76_2509	2666131..2669603	DR76_2506	*rob*	right origin-binding protein	2x
	2	252538	DR76_2120	DR76_2124	2270218..2276377	DR76_2121	*soxS*	regulatory protein SoxS	2x
	3	749	DR76_526	DR76_530	599986..602843	Not found			
	4	301	DR76_4346	DR76_4347	4565519..4569012	Not tested			
	5	421	DR76_256	DR76_257	275709..279004	Not tested			
	6	109	DR76_3955	DR76_3957	4149845..4153048	Not tested			
	7	451	DR76_690	DR76_690	766180..767439	Not tested			
TET	1	37626	DR76_2505	DR76_2509	2666131..2669603	DR76_2506	*rob*	right origin-binding protein	2x
	2	20050	DR76_2120	DR76_2124	2270218..2276377	DR76_2121	*soxS*	regulatory protein SoxS	2x
	3	3906	DR76_2926	DR76_2932	3143332..3149938	DR76_2929	*sdiA*	regulatory protein SdiA	2x
	4	228	DR76_764	DR76_764	848552..849955	Not tested			
	5	296	DR76_937	DR76_937	1035386..1037077	Not tested			
	6	427	DR76_1953	DR76_1953	2093599..2094783	Not tested			
	7	169	DR76_148	DR76_151	155500..163092	Not tested			
	8	1235	DR76_4795	DR76_4795	5072602..5074038	Not tested			
	9	132	DR76_3676	DR76_3681	3914999..3918737	Not tested			

	10	127	DR76_4370	DR76_4372	4589862..4591389	Not tested			
	11	879	DR76_470	DR76_470	530256..533155	Not tested			
	12	848	DR76_676	DR76_676	751405..751481	Not tested			
	13	169	DR76_923	DR76_923	1021553..1021852	Not tested			
	14	371	DR76_2044	DR76_2044	2181525..2183057	Not tested			
TMP	1	406354	DR76_2556	DR76_2564	2722303..2729487	DR76_2559	*folA*	dihydrofolate reductase	16x
	2	187	DR76_2507	DR76_2508	2666704..2668901	DR76_2506	*rob*	right origin-binding protein	1x
	3	244	DR76_690	DR76_690	766180..767439	Not tested			
	4	840	DR76_2724	DR76_2724	2910603..2910716	Not tested			

[#] The gene responsible for resistance is indicated. Not found, the plasmid could not be isolated from the plasmid pool; Not tested, the plasmid was not tested for resistance.

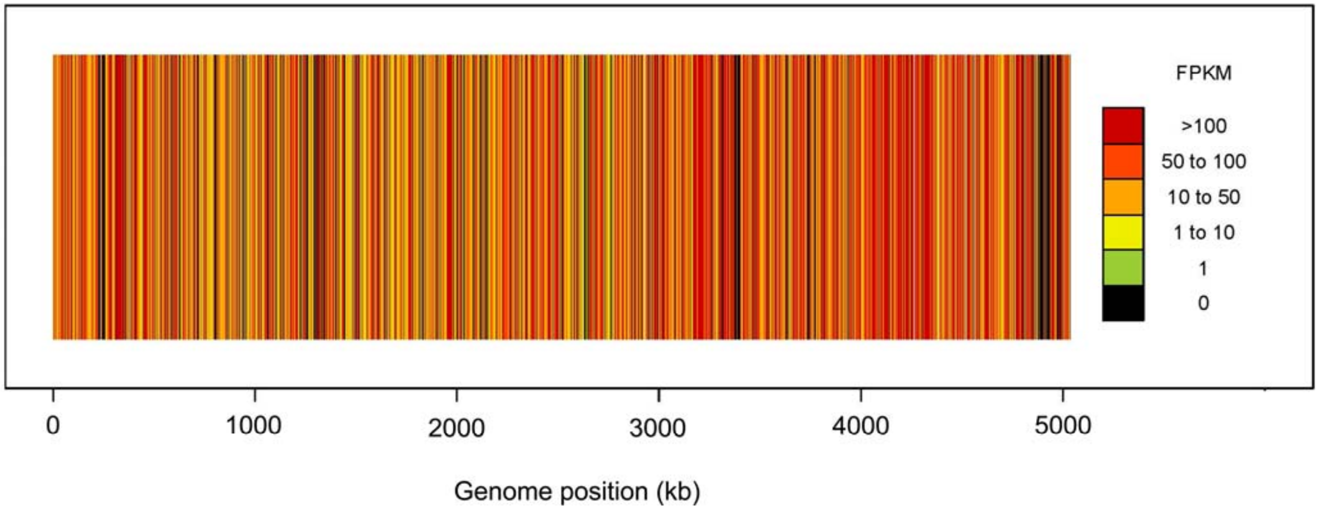

Figure S1. Baseline coverage of the *E.coli* plasmid library**.** The plasmid library was electroporated in *E.coli* TOP10 and grown for one passage before plasmids were extracted and sequenced. The chromosome map indicates the abundance of each gene expressed as fragments per kilobase per million fragments mapped (FPKM). Colored bars indicate the abundance of individual genes.

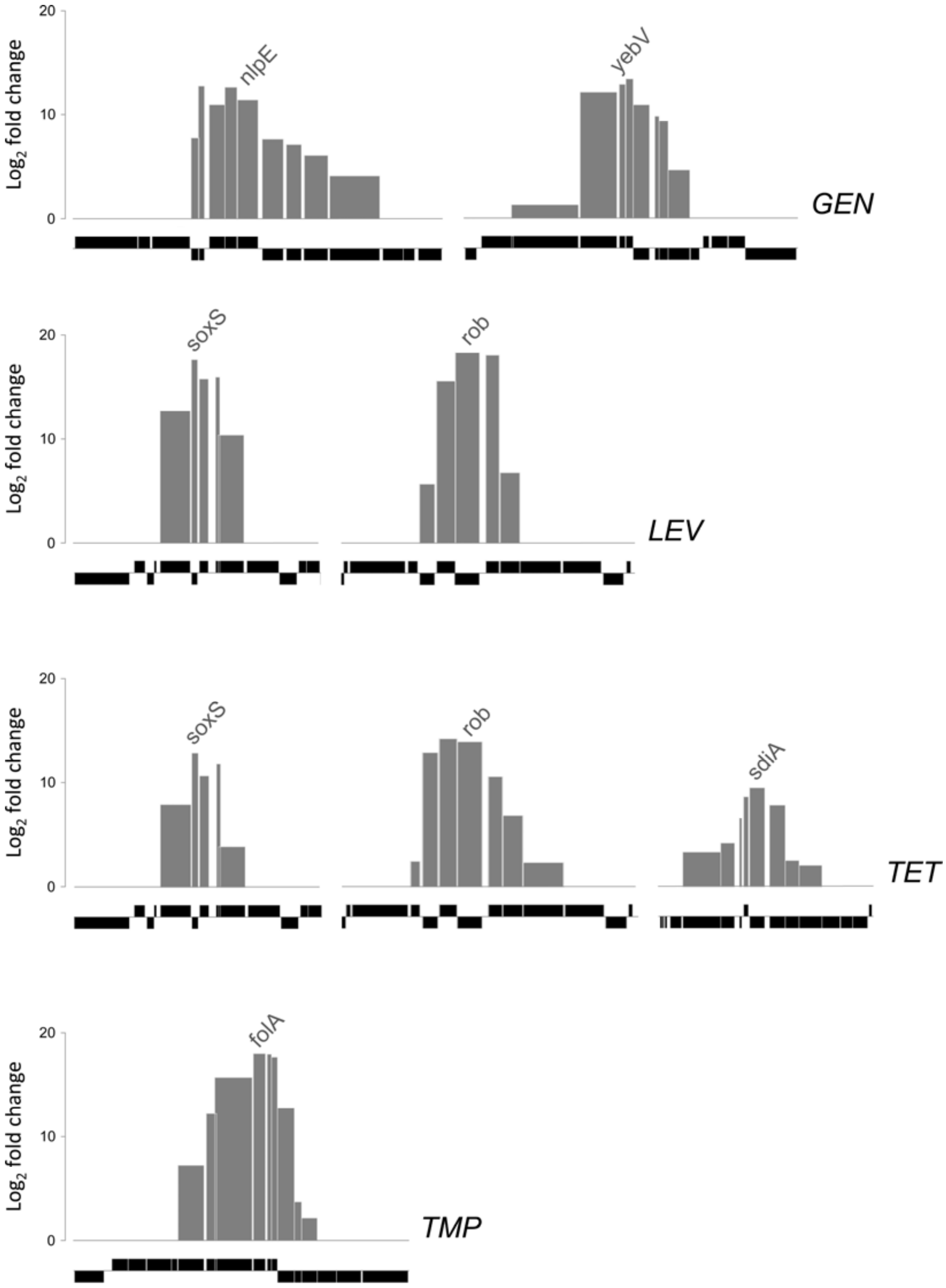

Figure S2. Gene enrichment for each plasmid enriched after gentamicin (GEN), levofloxacin (LEV), tetracycline (TET), and trimethoprim (TMP) selection that were functionally characterized. Log2-transformed maximal variation in abundance compared to the non-

treated baseline is shown. For each plasmid, the gene responsible for the resistance phenotype is indicated. Gray bars represent enriched genes. Black bars underneath represent operons (each box corresponding to a gene) surrounding the enriched loci.
